# Supplementary material for: Reduced Graphene Oxide Aerogel inside Melamine Sponge as an Electrocatalyst for the Oxygen Reduction Reaction
Source: Materials (Basel). 2021 Jan 9;14(2):322. doi: 10.3390/ma14020322 (PMC7827904; doi:10.3390/ma14020322)
Supplement: Supplementary file 1 [file materials-14-00322-s001.pdf]

SUPPORTING INFORMATION

Reduced Graphene Oxide Aerogel Inside Melamine Sponge as an Electrocatalyst for the Oxygen Reduction Reaction

Roman A. Manzhos [a], Sergey A. Baskakov [a], Evgeny N. Kabachkov [a, b], Vitaly I. Korepanov [c], Nadezhda N. Dremova [a], Yulia V. Baskakova [a], Alexander G. Krivenko [a], Yury M. Shulga [a, d], Gennady L Gutsev [e,*]

[a] *Institute of Problems of Chemical Physics, Russian Academy of Sciences, Chernogolovka 142432, Moscow Region, Russian Federation*

[b] *Chernogolovka Scientific Center, Russian Academy of Sciences, Chernogolovka 142432, Moscow Region, Russian Federation*

[c] *Institute of Microelectronics Technology and High Purity Materials, Russian Academy of Sciences, Chernogolovka 142432, Moscow Region, Russian Federation*

[d] *National University of Science and Technology MISIS, Leninsky pr. 4, Moscow 119049, Russian Federation*

[e] *Department of Physics, Florida A&M University, Tallahassee, Florida 32307, United States*

Experimental (GO synthesis)

For the GO synthesis, the modified Hummers method was used [1]. A vessel with a Teflon mixer and a thermometer was filled with 20 g of graphite powder and 650 ml of concentrated H_2SO_4. During the mixing of the load, 10 ml of concentrated HNO_3 were added, and the mixture was heated in a water heater at 45 °C. Next, it was cooled down to 10–15 °C, and 72 g of $KMnO_4$ were added gradually over 5 h while maintaining the temperature below 20 °C. The resulting mixture was heated to 40 °C, then mixed to a paste-like state and left for 24 hours. It was then cooled down to 10–15 °C, and 120 ml of water were slowly added at a temperature below 10–15°C. The mixture was kept at 45 °C for 1 h, then one litre of water was added when cooling down the mixture. The suspension obtained was then poured into a 3-litre glass vessel and slowly (in order to avoid foam formation) 70 ml of a concentrated H_2O_2 (28 mass%) were added. The suspension colour turned to bright yellow. The warm mixture was centrifuged, the deposit was dispersed in a 3% solution of HCl (2 litres) and centrifuged again. This operation was repeated 4 or 5 times. After the operation was completed, the GO sample was diluted with 25 litres of distilled water and washed until the acidity of the washing water was below 4 pH and no SO_4^{2-} and Cl^- ions remained. In order to prepare the water suspension of GO nanosheets, 300 mg of GO were mixed with 400 ml of water, followed by the suspension processing unit being placed in an ultrasonic bath for 2–4 h, and then finally centrifuging the suspension for 15 minutes at 3000 g.

Reference

[1] William, S.; Hummers, J. R.; Offeman, R. E. Preparation of Graphitic Oxide. *J. Am. Chem. Soc.* **1958**, *80* (6), 1339–1339.

Elemental Content of graphene oxide

The composition of graphene oxide depends on the method of their production. The graphene oxide selling by the Graphene Supermarket Company contains 79% of carbon and 20% of oxygen [1]. The remaining one percent left is likely contributed by hydrogen. In the pioneer work, Brodie [2] determined the graphite oxide composition C:O:H as 61.04:37.1:1.85 or, in molecular representation, as $C_{2.19}O_{1.00}H_{0.80}$. Our fresh samples of graphite oxide obtained using the Hummers method contained by mass 50.10% of carbon, 44.81% of oxygen and 2.69% of hydrogen. The sum of these quantities is somewhat smaller than

100% because the sample contains some technological impurities, which may reside in closed pores of graphite oxide and therefore cannot be washed out with distilled water.

References

[1] https://graphene-supermarket.com/Dispersion-in-Water-Single-Layer-Graphene-Oxide-175-ml.html.
[2] B. C. Brodie, On the Atomic Weight of Graphite. *Phil. Trans. R. Soc. London*, 149 (1859) 249–259.

X-Ray Photoelectron Spectra of Graphene Oxide

X-ray photoelectron spectroscopy [1] (XPS) is widely used for the characterization of carbon-based materials and graphene oxide in particular [2]. It is natural to consider that for a single-layer graphene oxide nanosheets composition determined by XPS, should coincide with the bulk composition of the film consisting of monolayer nanosheets. A survey spectrum of a GO sample presented by a film obtained by precipitation of GO suspension is presented in Figure *S*1. The oxygen concentration is estimated in 18 – 25 at. %, whereas concentrations of sulfur, nitrogen, and chlorine (technological impurities) are 0.6 – 1.3 at. %, > 0.3 at. % and > 0.2 at. %, respectively.

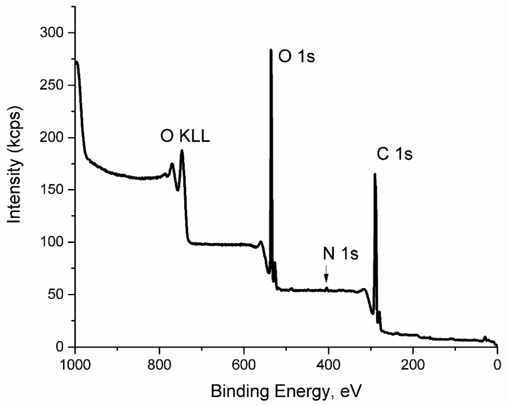

Figure *S*1. The survey XPS spectrum of a GO film. The Cl 2*p* peak is located at ~200 eV.

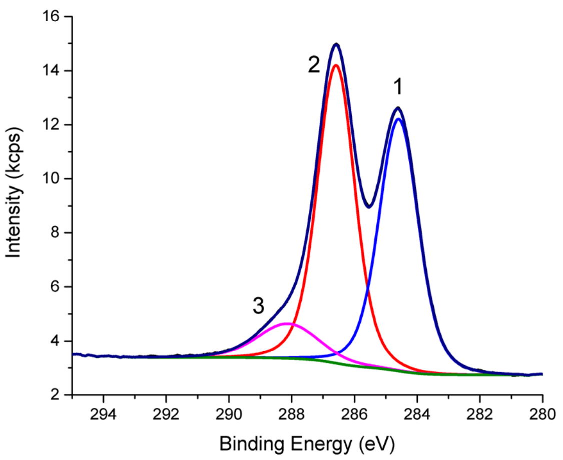

Figure *S*2. The C 1*s* XPS spectra of GO films and their decomposition.

High-resolution XPS C 1*s* core level spectra of GO films shown in Figure *S*2 contain three peaks (Table S1): peak 1 at 284.5 eV which is due to carbon atoms in the graphene network, peak 2 at 286.5 eV which is due

to carbon atoms singly boned with oxygen atoms, and peak 3 at 288.5 eV which is due to carbon atoms of carbonyl groups. This peak assignment is in agreement with assignments made elsewhere in the literature [3–7].

Table S1. The results of approximation of the XPS C1*s* spectrum of the GO film: C 1*s* binding energies (*BE*, in eV) and relative peak intensities (*I*, arbitrary units)

	Peak 1	Peak 2	Peak 3
BE	284.6	286.6 ± 0.1	288.5 ± 0.2
I	42.89	48.07	9.02

References

[1] *Practical surface analysis by Auger and X-ray photoelectron spectroscopy*. Eds. D. Briggs and M. P. Seah (John Wiley and Sons Ltd, Chichester, 1983.

[2] D. N. Voylov, A. L. Agapov, Y. M. Shulga, A. P. Sokolov, A. A. Arbuzov. Room temperature reduction of multilayer graphene oxide film on a copper substrate: Penetration and participation of copper phase in redox reactions. *Carbon*, 69 (2014) 563–570.

[3] Y. M. Shulga, T. C. Tien, C. C. Huang, S. C. Lo, V. E. Muradyan, N. V. Polyakova, Y. C. Ling, R. O. Loutfy, A. P. Moravsky, XPS study of fluorinated carbon multi-walled nanotubes. J. Electron Spectrosc. *Related Phenom*. 160 (2007) 22–28.

[4] D. Yang, A. Velamakanni, G. Bozoklu, S. Park, M. Stoller, R. D. Piner, S. Stankovich, I. Jung, D. A. Field, C. A. Ventrice Jr., Ro. S. Ruoff, Chemical analysis of graphene oxide films after heat and chemical treatments by X-ray photoelectron and Micro-Raman spectroscopy. *Carbon*, 47 (2009) 145–152.

[5] H. Shin, K. Kim, A. Benayad, S. Yoon, H. Park, I. Jung, M. Jin, H. Jeong, J. Kim, J. Choi, Y. Lee, Efficient Reduction of Graphite Oxide by Sodium Borohydride and Its Effect on Electrical Conductance. *Adv. Funct. Mater*. 19 (2009) 1987–1992.

[6] S. Pei, H. Cheng, The reduction of graphene oxide. *Carbon,* 50 (2012) 3210–3228.

[7] G. Sobon, J. Sotor, J. Jagiello, R. Kozinski, M. Zdrojek, M. Holdynski, P. Paletko, J. Boguslawski, L. Lipinska, K. M. Abramski, Graphene Oxide vs. Reduced Graphene Oxide as saturable absorbers for Er-doped passively mode-locked fiber laser. *Opt. Express*, 20 (2012) 19463–19473.

Raman Spectra of Graphene Oxide

Raman spectra are often invoked for characterization of various carbon compounds. The Raman spectrum of a diamond contains [1–4] a narrow peak at 1332 cm^{-1}, whereas the most intense peak in Raman spectra of “good” graphite, e.g., highly-oriented pyrolytic graphite (HOPG), usually denoted as the peak G is located [5] at 1580 cm^{-1}. If graphite has a large number of defects, then the peak D named as a disorder peak appears [6–11] at ~1350 cm^{-1}. Raman spectrum of graphene oxide under study is shown in Figure 3 and the peak energies are presented in Table 2. The peak designations therein correspond to the designations commonly used in the literature.

The ratio intensities I_D/I_G of peaks G and D, can be related [4] to graphene crystallite sizes in the basal plane L_a according to the following equation:

$$L_a = (2.4 \times 10^{-10})\ \lambda^4_L\ (I_D/I_G)^{-1} \quad (1)$$

where λ_L is the laser wavelength in nm. When computed according to Eq. 1 sizes L_a of the sample, whose I_D/I_G ratio is given in Table 2, is 20 nm.

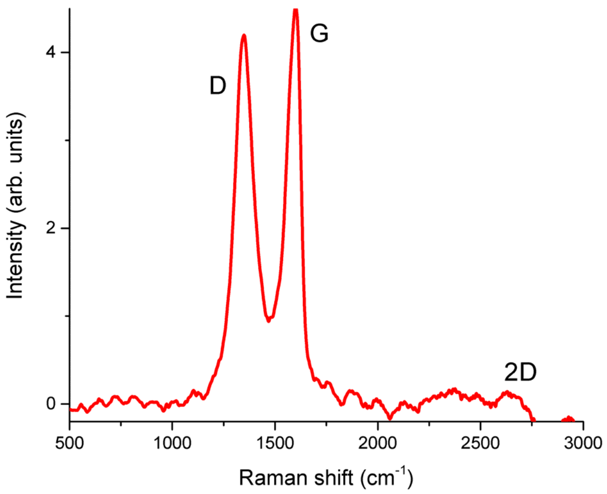

Figure S3. A Raman spectrum of GO. The excitation wavelength was 532 nm.

Table S2. Positions of the peak maxima and ratios of integral intensities of the major bands in the Raman spectra of GO samples obtained by different research groups

D band	G band	Intensity ratio,	λ,[a] nm	Reference
Peak, cm-1	Peak, cm-1	I_D/I_G		
1334	1582	1.16	633	[7]
1350	1595	…	633	[8]
1347	1603	1.2	532	[9]
1363	1594	…	514	[10]
1375	1588	…	514	[11]
1348	1600	0.95	532	This work

[a] λ is the laser wavelength.

References

[1] S. Prawer, K. W. Nugent, D. N. Jamieson, J. O. Orwa, L. A. Bursill, J. L. Peng, The Raman spectrum of nanocrystalline diamond. *Chem. Phys. Lett*. 332 (2000) 93–97.

[R] J. Nemanich, J. T. Glass, G. Lucovsky, R. E. Shroder, Raman scattering characterization of carbon bonding in diamond and diamondlike thin films. *J. Vac. Sci. Technol. A*, 6 (1988) 1783-1787.

[3] D. S. Knight, W. B. White, Characterization of diamond films by Raman spectroscopy. *J. Mater. Res.* 4 (1989) 385-393.

[4] M. A. Pimenta, G. Dresselhaus, M. S. Dresselhaus, L. G. Cancado, A. Jorio, R. Saito, Studying disorder in graphite-based systems by Raman spectroscopy. *Phys. Chem. Chem. Phys.* 9 (2007) 1276–1291.

[5] L. G. Cancado, M. A. Pimenta, B. R. A. Neves, M. S. S. Dantas, A. Jorio, Influence of the Atomic Structure on the Raman Spectra of Graphite Edges. *Phys. Rev. Lett.* 93 (2004) 24740.

[6] S. Hong, S. Jung, S. Kang, Y. Kim, X. Chen, S. Stankovich, S. R. Ruoff, S. Baik, Dielectrophoretic deposition of graphite oxide soot particles. *J. Nanosci. Nanotechnol.* 8 (2008) 424-427.

[7] G. Wang, J. Yang, J. Park, X. Gou, B. Wang, H. Liu, J. Yao, Facile Synthesis and Characterization of Graphene Nanosheets. *J. Phys. Chem. C*, 112 (2008) 8192-8195.

[8] Y. Matsumoto, M. Koinuma, S. Y. Kim, Y. Watanabe, T. Taniguchi, K. Hatakeyama, H. Takiishi, H. S. Ida, Simple Photoreduction of Graphene Oxide Nanosheet under Mild Conditions, *Appl. Mater. Interfaces*, 2010, 2, 3461-3466.

[9] S. Stankovich, D. A. Dikin, R. D. Piner, K. A. Kohlhaas, A. Kleinhammes, Y. Jia, Y. Wu, S. B. T. Nguen, S. R. Ruoff, Synthesis of graphene-based nanosheets via chemical reduction of exfoliated graphite oxide. *Carbon*, 45 (2007) 1558–1565.

[10] T. S. Sreeprasad, A. K. Samal, T. Pradeep, Tellurium Nanowire-Induced Room Temperature Conversion of Graphite Oxide to Leaf-like Graphenic Structures. *J. Phys. Chem. C*, 113 (2009) 1727-1737.
[11] K. N. Kudin, B. Ozbas, H. C. Schniepp, R. K. Prud'homme, I. A. Aksay, R. Car, Raman spectra of graphite oxide and functionalized graphene sheets. *Nano Lett.* 8 (2008) 36–41.

3.1. GO sheet and film morphology

The dimensions of GO nanosheets depend significantly on the initial graphite, the duration and temperature of subsequent washing, drying and storage operations. Figure 4 shows SEM images of individual large sheets of exhaust gas on a smooth surface.

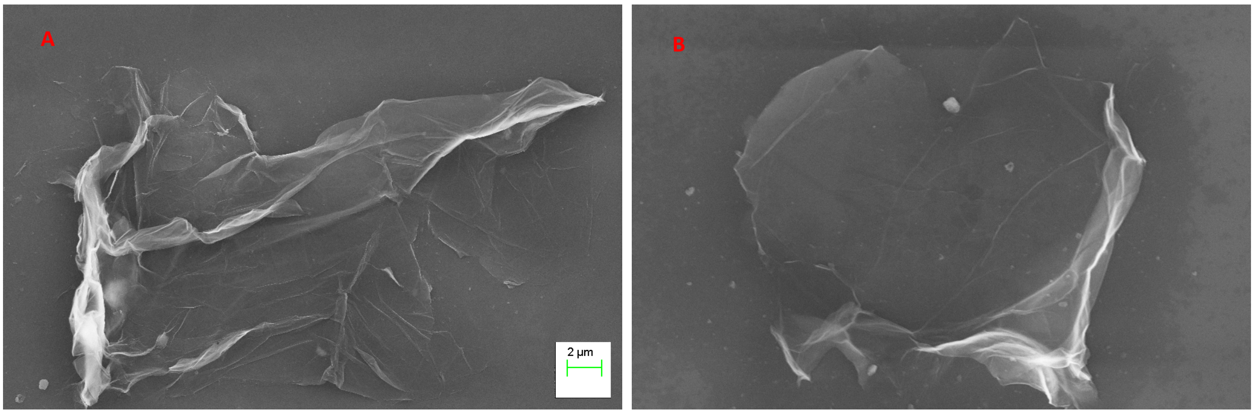

Figure *S*4. Microphotographs of GO nanosheets.

Thick GO films separated from substrates are sometimes named as graphene oxide papers (GOP). A microphotograph of both GOP sides is presented in Figure S5. It follows from Figure S6, which displays the GOP cross-section, that the paper structure is layered. The GO nanosheets were found to possess inhomogeneous packing because of the folds.

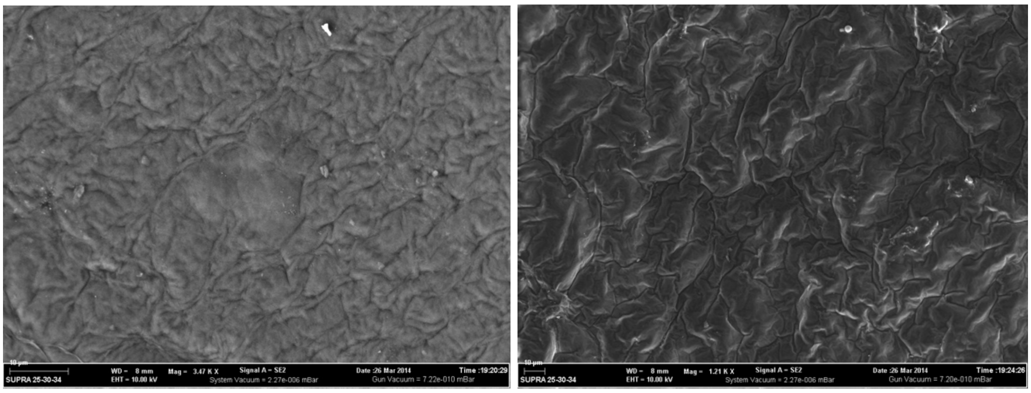

A B

Figure *S*5. Microphotographs of GO paper: A corresponds to the bright side; B corresponds to the matt side.

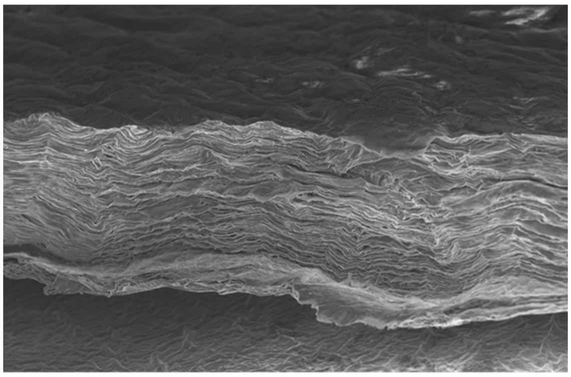

Figure *S*6. Microphotograph of a GO film cross section. The film is cut to the half of its width. The matt film surface is seen in the upper part of the microphotograph. The middle portion corresponds to the cross-section, whereas the bottom shows the inner layer of the film.

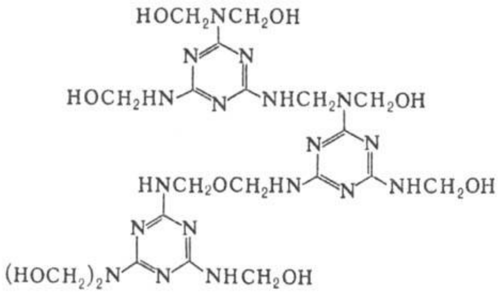

Figure *S*7. Melamine-formaldehyde (chemical formula)

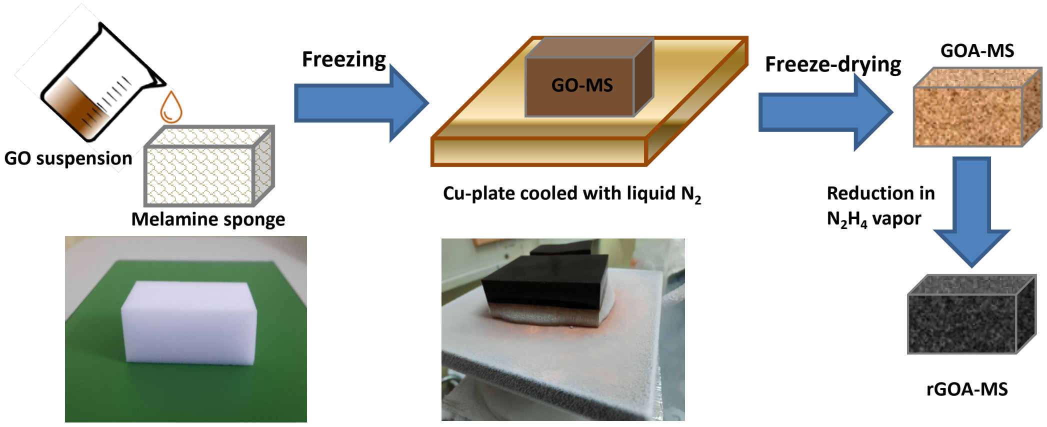

Figure *S*8. A scheme of obtaining rGOA-MS composites

Table *S*3. Results of C 1*s* Spectrum Decomposition for Composite Aerogel rGOA-MS

Peak	Binding energy, eV	Relative intensity, %	Assignment
1	284.6	69.88	C -C
2	285.9	18.11	C-N
3	286.7	6.72	C-O
4	287.8	3.41	C=O
5	288.9	1.88	O-C=O

Table *S*4. Results of N 1*s* Spectrum Decomposition for Composite Aerogel rGOA-MS

Peak	Binding energy, eV	Relative intensity, %	Assignment
1	398.9	10.63	pyridine-like (N1)
2	399.8	83.81	pyrrole (5-membered cycle) (N2)
3	400.9	2.73	graphite-like N (N3)
4	402.2	1.73	graphite-like N (N4)
5	402.9	1.09	oxidized pyridine N (N5)
